# Newly Diagnosed Multiple Sclerosis Presenting as Brown-Séquard Syndrome: A Case Report

**DOI:** 10.5811/cpcem.2022.2.55317

**Published:** 2022-05-06

**Authors:** Shelby Hoebee, Levi Howard, James Komara, Megan McElhinny

**Affiliations:** * Creighton University Arizona Health Education Alliance, Valleywise Health, Department of Emergency Medicine, Phoenix, Arizona; † Mayo Clinic Arizona, Department of Emergency Medicine, Phoenix, Arizona

**Keywords:** Brown-Séquard syndrome, case report, multiple sclerosis

## Abstract

**Introduction:**

Brown-Séquard syndrome is a rare neurological disorder due to hemisection of the spinal cord that can occur from a variety of causes, most commonly trauma.

**Case Report:**

We present a case of a 25-year-old woman presenting with Brown-Séquard syndrome as her first clinical presentation of multiple sclerosis.

**Conclusion:**

This case highlights the need to have demyelinating disease on the differential as an exceedingly rare, but important, possible cause of Brown-Séquard syndrome.

## INTRODUCTION

Brown-Séquard syndrome (BSS) is a neurological disorder caused by hemisection of the spinal cord. It is characterized by ipsilateral upper motor neuron motor weakness below the level of lesion, lower motor neuron type at the level of lesion, and loss of ipsilateral proprioception with contralateral loss of pain and temperature sensations below the level of lesion.^1^ The syndrome was first described by Charles-Édouard Brown-Séquard in 1849 through his experiments with animal models.^2^ Most commonly, BSS is caused by trauma such as stab wounds and is occasionally caused by tumors, with lesser known causes including degenerative diseases, infection, and ischemia.^3^

Brown-Séquard syndrome is considered an incomplete spinal cord injury. Hemisection of the spinal cord resulting in classic presentation is much more rare than partial hemisection.^4^ Therefore, clinical presentation of BSS can range from mild to severe neurological deficits depending on the degree of hemisection. We report a case of an undiagnosed multiple sclerosis in a 25-year-old female that presented with BSS.

## CASE REPORT

A 25-year-old, right-hand dominant, White female with no significant past medical history presented to the emergency department with complaints of one week of right lower extremity numbness from her toes to her hip and three days of left lower extremity weakness. The patient’s weakness had been progressively getting worse to the point of using crutches because she was unable to pick up her left foot. She stated that she fell from standing height onto her left side, landing on a fully inflated air mattress while playing with her daughter three days before her numbness started, but reported no other traumatic injuries. She stated that the fall was minor and she didn’t think much of it. She stated that she went to a chiropractor two days after developing the right leg numbness without any significant improvement or worsening of her symptoms. The patient denied fevers, chill, facial weakness, numbness or paresthesias in upper extremities, difficulty swallowing, changes in speech, or visual symptoms.

On examination, the patient was alert and oriented to person, place, time, and situation. She was conversant, and her speech production and comprehension as well as cranial nerves were all normal. Visual fields were fully intact with no evidence of nystagmus or extraocular muscle weakness. Motor exam revealed complete, symmetrical strength, reflexes, and sensation in bilateral upper extremities. Lower extremity exam was significant for 3/5 strength in left hip flexion, 4/5 in left knee extension, 3/5 strength in left foot dorsiflexion, and 5/5 strength in left plantarflexion. The right leg showed full 5/5 strength in the previously mentioned muscle groups.

Following these exam findings, neurology was consulted with confirmation of the above findings as well as additional sensory deficits. Their exam showed right lower extremity with decreased pinprick and temperature sensation up to the thoracic (T) 11-T12 dermatomes. Left lower extremity had diminished proprioception and vibration with dysesthesia from the mid-shin down, but she was able to feel temperature and pinprick. The patient had sustained clonus at the left patella with 6–7 beats on the right patella. Ankle jerk was normal and symmetric. Babinski reflex was equivocal with significant withdrawal and negative Hoffman sign. Coordination was intact.

**Image 1:**
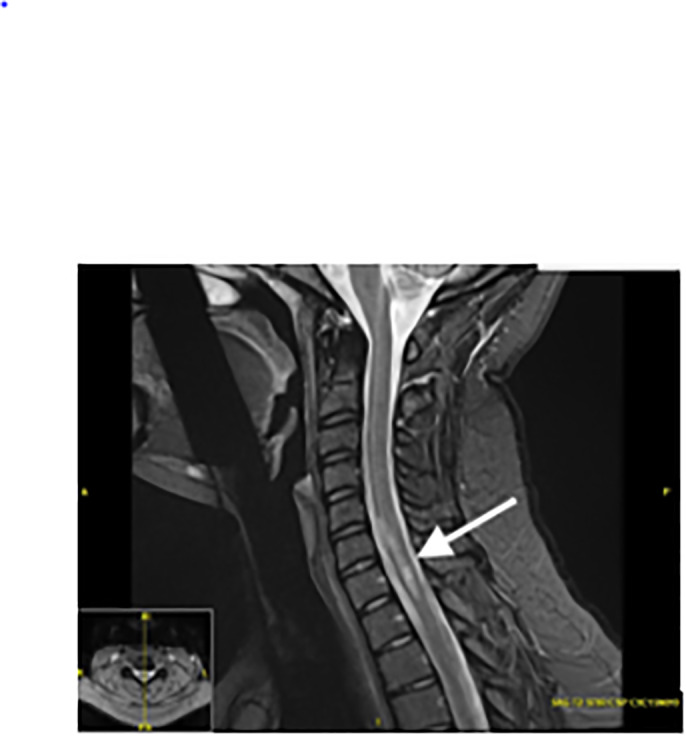
Sagittal T2-weighted magnetic resonance imaging with asymptomatic hyperintensity within the cervical spine, worse at cervical level 5–7 (arrow).

**Image 2:**
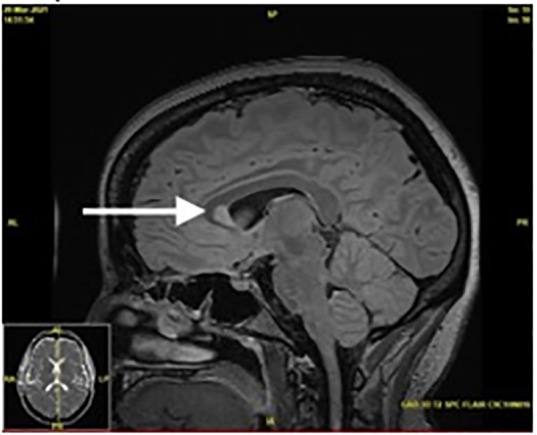
Sagittal T2-weighted magnetic resonance imaging showing periventricular hyperintensity characteristic of multiple sclerosis.

A magnetic resonance imaging (MRI) of the patient’s brain, cervical, thoracic, and lumbar spine was completed to evaluate for the cause. Imaging showed multifocal areas of T2 hyperintensity throughout the spine with contrast enhancement, suspicious for a demyelinating or inflammatory disease without space-occupying lesions. The patient had large regions of active demyelination in the mid and lower cervical spine with nearly all levels involved. Although greatest areas of demyelination spanned from the fifth to seventh cervical vertebrae, these areas were asymptomatic (Image 1). Magnetic resonance imaging of the head showed periventricular, juxtacortical, and infratentorial enhancing lesions, typical of multiple sclerosis (Image 2).

**Image 3:**
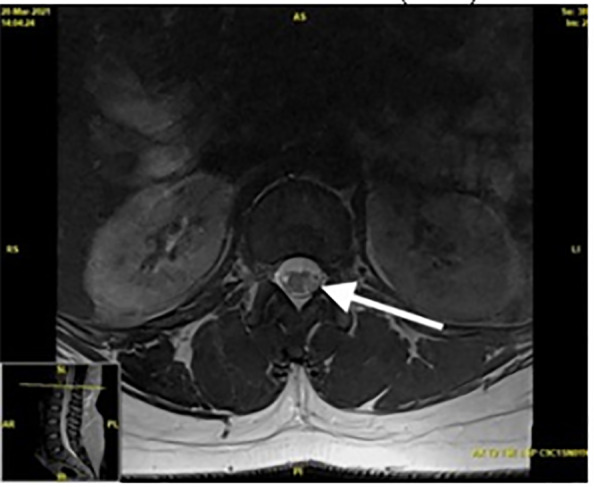
Axial T2-weighted magnetic resonance imaging showing a high signal intensity within the left spinal cord at the level of the first lumbar vertebra (arrow).

Axial T2-weighted MRI images showed high signal intensity hemisecting the spinal cord at a level of the first lumbar vertebra, likely leading to her symptoms of BSS (Image 3). Following the above findings, a diagnosis of Brown-Séquard syndrome was made and the patient was admitted for further workup as to the etiology of her neurologic deficits. Upon admission, the patient received further laboratory studies including workup for tuberculosis, John Cunningham virus, human immunodeficiency virus, extractable nuclear antigen antibody, antinuclear antibody, hepatitis C, neuromyelitis optica (NMO), hepatitis B, autoimmune encephalopathy, and kappa free light chains, all of which were within normal limits. The patient’s lumbar puncture revealed a lymphocytic pleocytosis.

As treatment, the patient received one gram of intravenous (IV) methylprednisolone for seven days with near complete recovery of strength by day three. She was discharged with mild residual numbness, but otherwise had a complete recovery. She was placed on outpatient IV ocrelizumab every six months as a disease-modifying agent.

**CPC-EM Capsule**

What do we already know about this clinical entity?

*Brown-Séquard syndrome is caused by hemi-section of the cord and characterized by a unique distribution of upper and lower motor neurons symptoms*.

What makes this presentation of disease reportable?

*This is a reportable presentation because it is an extremely rare etiology of Brown-Séquard*.

What is the major learning point?

*It is important to keep demyelinating disorders on the differential as a potential cause of Brown-Séquard*.

How might this improve emergency medicine practice?

*Recognizing multiple sclerosis as the cause of Brown-Séquard can change management and highlight the need for a broad differential even in rare presentations*.

## DISCUSSION

Brown-Séquard syndrome is caused by damage to the ipsilateral corticospinal tracts and dorsal column as well as the contralateral spinothalamic tract. This results in the ipsilateral loss of strength and proprioception as well as contralateral loss of temperature and pinprick sensation.^5^ Most commonly, patients present with a partial BSS due to a partial hemisection; pure BSS is extremely rare.

Our patient presented with a subacute onset of symptoms that were suggestive of BSS, and she was later confirmed to have a spinal cord lesion causing hemisection. Aside from the minor fall the patient had described, there was no other evidence or history of significant trauma. The symptoms of acute BSS typically occur immediately following traumatic injuries.^6^ Therefore, the patient’s fall was likely coincidental in timing and largely unrelated to the development of her symptoms. Imaging ruled out etiologies such as cervical disc herniation, epidural hematoma, or syringomyelia as a cause. Lesser known causes such as NMO or infection were ruled out through negative blood and cerebrospinal fluid (CSF) studies. Given the patient’s classic findings of multiple sclerosis (MS) on both her head and spinal MRIs, steroids were started. This treatment resulted in significant resolution of her symptoms, confirming our suspicion of MS.

Multiple sclerosis is an autoimmune, chronic inflammatory disorder that predominately affects White females aged 20–40 with symptoms varying in involvement of sensory, motor, visual, and brainstem pathways.^7^ The diagnosis of MS is clinical, but supportive evidence can be found through modalities such as MRI or CSF analysis. Multiple sclerosis is diagnosed by at least two typical attacks or by a single typical demyelinating event combined with evidence of dissemination in space or time by MRI.^7^ Dissemination in space (DIS) is defined as one demyelinating lesion in at least two of four areas (periventricular, juxtacortical, infratentorial, and spinal). Dissemination in time (DIT), on the other hand, is defined as simultaneous presence of asymptomatic gadolinium-enhancing and non-enhancing lesions at any time.^7^ Although it was our patient’s first known attack of MS, she had evidence of both DIT and DIS lesions on her MRI.

Upon reviewing the literature, we found that MS is one of the rarest known causes of BSS with very few case reports in existence.^1^,^8^,^9^,^10^ The exact incidence of BSS due to MS could not be found, likely secondary to its relative rarity. Occasionally, BSS presents as a complication in a patient with known MS,^11^ but BSS as an initial presentation is even more rare.

## CONCLUSION

Our patient presented with Brown-Séquard Syndrome as her first and only presentation of a new diagnosis of multiple sclerosis. We report this case due to the rarity of presentation and to record that a demyelinating disorder can initially present as an atypical neurological condition such as Brown-Séquard syndrome.
